# Supplementary material for: MS785-MS27 Reactive Misfolded/Non-Native Zn-Deficient SOD1 Species Exhibit Cytotoxicity and Adopt Heterozygous Conformations in Motor Neurons
Source: Int J Mol Sci. 2024 May 21;25(11):5603. doi: 10.3390/ijms25115603 (PMC11171496; doi:10.3390/ijms25115603)
Supplement: Supplementary file 1 [file ijms-25-05603-s001.zip › Supplementary text_ijms-2973322.pdf]

Figure S1 Recognition of SOD1 species by MS785 or MS27 alone

(**A**, **B**) Indirect ELISA using the metal-binding and conformation-disordered WT SOD1 species analyzed with (**A**) MS785 alone and (**B**) MS27 alone. Apo-WT SOD1^{SH} was used as an internal control. All data are given as the mean ± SD (n = 4 per group). The significance of differences was analyzed using one-way ANOVA followed by Tukey–Kramer's *post hoc* test. **P<0.01 (vs. apo-WT SOD1^{SH}). N.S. = not significant. (**C**) Indirect ELISA with (*left*) MS785 alone, (*middle*) MS27 alone, and (*right*) the MS785-MS27 cocktail for apo-G37R SOD1^{SH}. Apo-WT SOD1^{SH} was used as a positive control for each antibody. Data are given as the mean ± SD (n = 4 per group).

Figure S2 Recognition of murine SOD1 species by MS785-MS27 antibody cocktail

(**A**) A representative image showing Instant Blue Coomassie staining with murine WT SOD1 proteins. For analysis of SOD1 with a disulfide bond, the protein at 10 μM was treated with 40 mM iodoacetamide at 37ºC for 1 h and subjected to SDS-PAGE under non-reducing conditions. SH = disulfide bond-cleaved SOD1; S-S = disulfide bond-formed SOD1. Indirect ELISA with (**B**) the MS785-MS27 cocktail and (**C**) MS27 alone for murine SOD1. Human apo-WT SOD1^{SH} was used as an internal control. Data are given as the mean ± SD (n = 4 per group). (**D**) Comparisons of the amino acid sequences between murine SOD1 and human SOD1 in the epitope regions of MS785 and MS27.
