# Supplementary figures and images for: MS785-MS27 Reactive Misfolded/Non-Native Zn-Deficient SOD1 Species Exhibit Cytotoxicity and Adopt Heterozygous Conformations in Motor Neurons
Source: Int J Mol Sci. 2024 May 21;25(11):5603. doi: 10.3390/ijms25115603 (PMC11171496; doi:10.3390/ijms25115603)

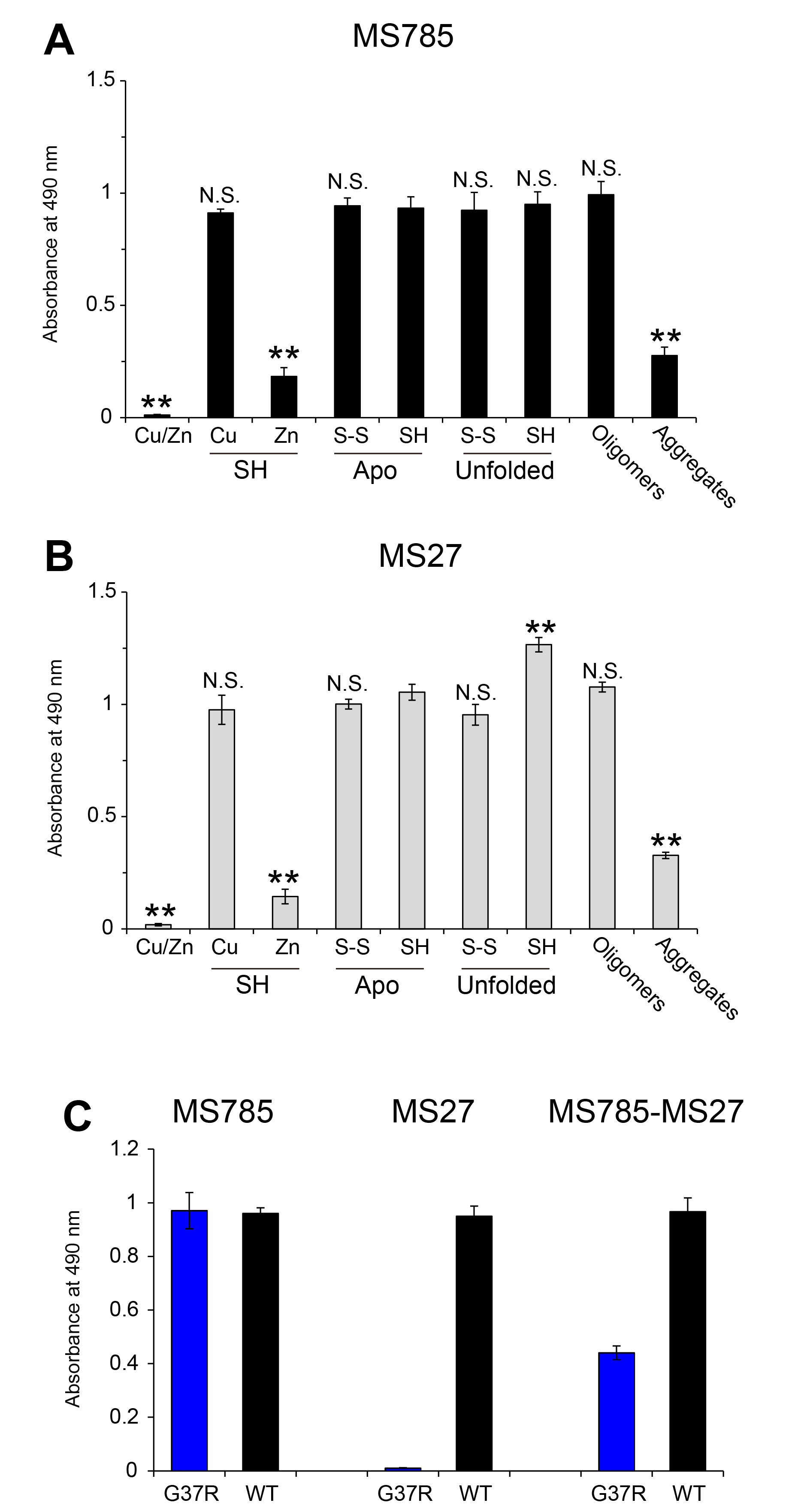

Supplement: Supplementary file 1 [file ijms-25-05603-s001.zip › Figure S1.tif]

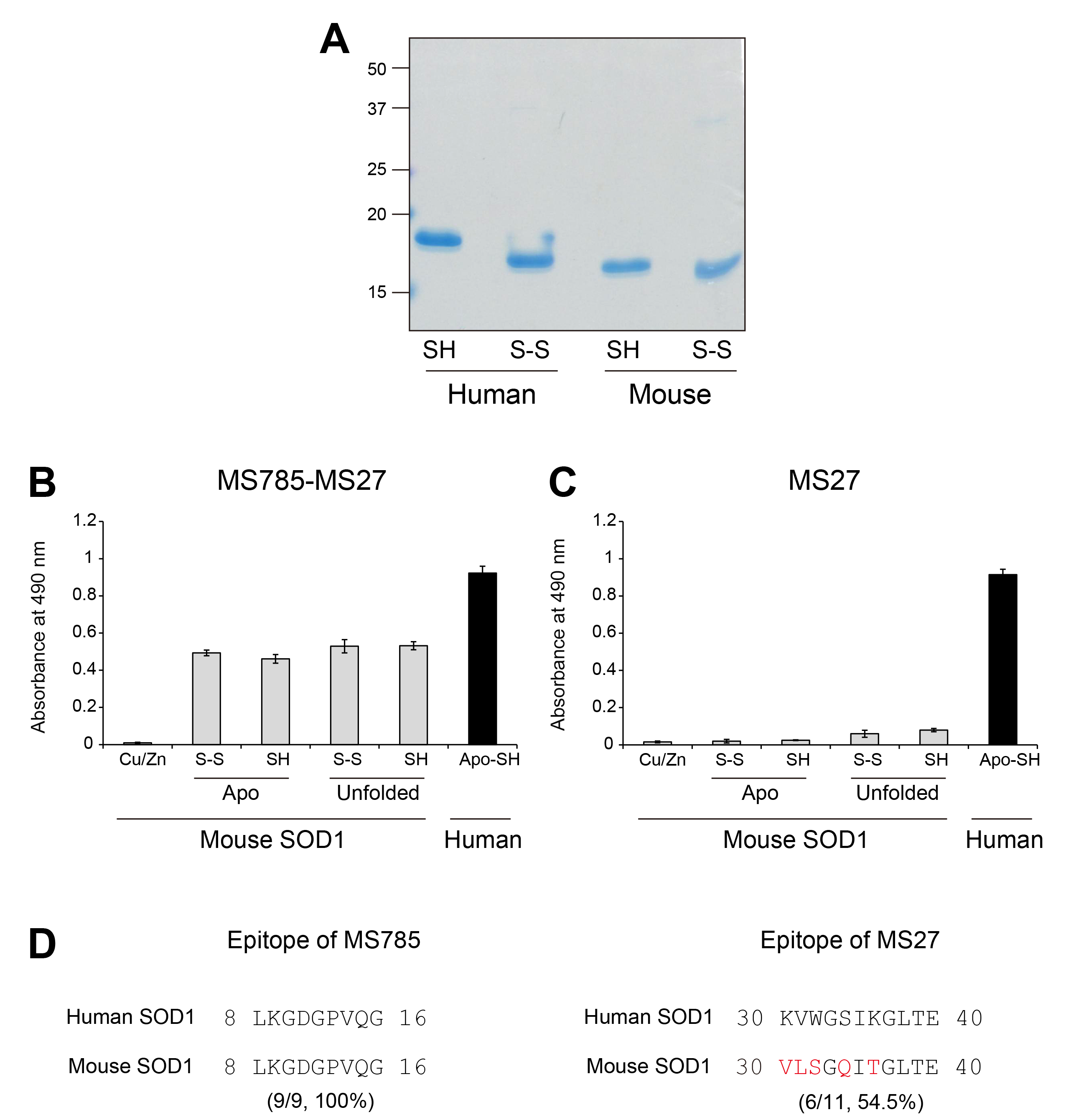

Supplement: Supplementary file 1 [file ijms-25-05603-s001.zip › Figure S2.tif]
